# Circ-Usp10 promotes microglial activation and induces neuronal death by targeting miRNA-152-5p/CD84

**DOI:** 10.1080/21655979.2021.2004362

**Published:** 2021-12-07

**Authors:** Dake Tong, Yanyin Zhao, Yang Tang, Jie Ma, Zhiwei Wang, Cheng Li

**Affiliations:** a Shanghai Key Laboratory of Orthopaedic Implants, Department of Orthopaedic Surgery, Shanghai Ninth People’s Hospital, Shanghai Jiaotong University School of Medicine, Shanghai, People’s Republic of China; b Department of Neurology, Huashan Hospital, Fudan University, Shanghai, China; c Department of Orthopedic Surgery, The Third Affiliated Hospital of Naval Medical University, Shanghai, China; d Department of Orthopedics, Changhai Hospital, Naval Medical University, Shanghai, P.R. China

**Keywords:** Spinal cord injury (SCI), microglial activation, neuronal death, circ-Usp10, miR-152-5p

## Abstract

Spinal cord injury (SCI) is a traumatic disease resulting in neuronal injury. circRNAs are closely associated with human diseases. Nevertheless, the potential mechanism by which circRNAs impact SCI remains to be elucidated. The aim of this study was to investigate the regulatory roles of Circular RNAs (circRNAs) in SCI. The SCI mouse model and integrated bioinformatics analysis were used to identify the differentially expressed genes (DEGs). Functional enrichment analysis was conducted to study the related pathways. The circRNA-mediated ceRNA network and subnetwork was constructed based on circMir, TargetScan and miRanda. qRT–PCR, ELISA, flow cytometry, and luciferase reporter assays were carried out to validate the role of circ_0014637 (circ-Usp10) and microRNA(miR)-152-5p /CD84 in microglia. In all, 23 DE-circRNAs, 127 DE-miRNAs and 1327 DE-mRNAs were identified. We integrated these DEGs to construct a circRNA-miRNA-mRNA network. The circ-Usp10/miR-152-5p/CD84 axis was found to function in microglial activation. We also found that circ-Usp10 inhibited the secretion of proinflammatory factors in microglial BV2 cells. In addition, silencing circ-Usp10 significantly reduced the death of the neuronal cell line HT22. Taken together, we concluded that circ-Usp10 may function as a competing endogenous RNA (ceRNA) to promote microglial activation and induce neuronal death by targeting miR-152-5p/CD84. The circ-Usp10 may be a diagnostic biomarker and potential target for SCI therapy.

## Introduction

Spinal cord injury (SCI) is a traumatic disease of the central nervous system that elicits chronic pain in 65% of individuals [1–4]. In addition, SCI afflicts an increasing number of aged individuals and has become a burden with heavy economic and social costs worldwide [5,6]. Due to the complex pathologic conditions, novel treatm.ent remains an area of significant unmet medical need because of limited effective targets [7–10]. The current treatment of SCI is mainly focused on improving the inflammatory microenvironment and the regeneration and repair of nerve function. Although there has been considerable progress in understanding the mechanism of pain, widespread lipid peroxidation, cell apoptosis and severe inflammation create a terrible environment for nerve axonal regeneration [11,12]. Hence, it is of great significance to explore novel treatments and therapeutic targets for SCI.

Circular RNAs (circRNAs) are a class of conserved endogenous noncoding RNAs that are involved in transcriptional and posttranscriptional gene regulation and are highly enriched in the nervous system [13]. circRNAs can act as competing endogenous RNAs (ceRNAs) and compete for microRNAs (miRNAs) with other RNAs through miRNA response elements (MREs), which affect the regulation of target genes by miRNAs and thereby affect the progression of diseases [14]. They participate in the survival and differentiation of multiple nerve cells and may even promote the recovery of neurological function after stroke. However, their role in the inflammatory response after spinal cord injury remains unclear. In recent years, some circRNAs and ceRNAs have been reported to affect the SCI process. For example, Wang et al. indicated that circRNA Plek promotes fibrogenic activation by regulating the miR-135b-5p/TGF-βR1 axis after SCI [15]. However, the roles of the circRNA-mediated ceRNA network in the pathogenesis of SCI require further investigation.

The above research suggests that further study of the function of the ceRNA network based on circRNAs to encode proteins is an effective strategy for understanding SCI.

In this study, we aimed to construct a circRNA-mediated ceRNA network and investigate the related functions, pathways and underlying mechanisms. We performed RNA sequencing (RNA-Seq) of mRNA, miRNAs, and circRNAs based on the SCI mouse model. Then, integrated bioinformatics analysis was carried out to screen out the differentially expressed genes (DEGs). Functional enrichment analysis was conducted to study the related pathways. Furthermore, a circRNA-mediated ceRNA network was constructed, and a subnetwork including circ_0014637 (circ-Usp10) that regulates microglial/macrophage activation was screened. We found that circ-Usp10 inhibited the secretion of proinflammatory factors in microglial BV2 cells. In addition, circ-Usp10 silencing significantly reduced the death of the neuronal cell line HT22. These results revealed that circ-Usp10 is a potential biomarker and therapeutic target in SCI. This study may provide new clues for studying the mechanisms underlying SCI and present novel molecular targets for the clinical therapy of SCI in humans.

## Materials and methods

### Animals and the spinal cord injury model

A total of 9 three-month-old male C57BL/6 mice weighing 18 to 22 g were utilized. Mice were maintained for at least 7 days before the experiment in a temperature-regulated (23–25°C) and humidity-controlled (50% relative humidity) room on a 12-hour light/dark cycle. Food was withheld, but the mice were allowed free access to water overnight prior to surgery. All experiments were performed in accordance with the guidelines of the Animal Ethics Committee of the Second Military Medical University (Shanghai, China). The procedures for a complete transection SCI in mice have been previously described in detail [16]. In brief, to reveal the changes in gene expression levels of mice after SCI, we used C57BL/6 mice to construct a SCI group and a sham operation group containing 3 mice each. The mice were anesthetized with an intraperitoneal injection of pentobarbital (1%, 35 mg/kg) [17]. After the mice were anesthetized and depilated, the subcutaneous tissue and the muscle tissue on the spine extending from T8 to T10 of the SCI group were incised to reveal the T9 laminae. Then, hemisection was performed with an ophthalmic iris knife at the right T9. A MASCIS impactor was used, and the exposed dorsal surface of the cord was subjected to the impact of a dropped weight using a 10 g rod released from a height of 25 mm. The sham-operated animals received the same surgical procedures, but no impact was applied to the spinal cord [18,19]. All experiments were repeated three times.

### RNA sequencing and analysis

Total RNA including mRNA, miRNA was isolated from BV2 cells using TRIzol Reagent (TransGen Biotech). The T9 spinal cord tissues of the mice with spinal cord injury and the mice that underwent a sham operation were humanely removed for RNA-seq at 3 days. circRNA and miRNA sequencing and database construction were performed by Illumina HiSeq 2500. Single-end sequencing (<50 bp) was performed using an Illumina HiSeq 2500 high-throughput sequencing system. For lncRNA library preparation, paired-end sequencing (>200 bp) was performed on an Illumina HiSeq 4000 system.

### Bioinformatics analysis

GEO2R, which is an interactive web tool, was used to identify DEGs. After the RNA-seq data of the SCI and sham operation groups were normalized, the differential expression of mRNA (DE-mRNA), circRNAs (DE-circRNAs) and miRNAs (DE-miRNAs) was analyzed using the R package DEseq2 [20] with the thresholds of adjusted P value < 0.05 and fold change ≥ 2. Gene Ontology (GO) annotations [21] and Kyoto Encyclopedia of Genes and Genomes (KEGG) pathway analysis [22] were performed to investigate the roles of all DE-mRNAs. GO enrichment analysis, including biological process (BP), cellular component (CC) and molecular function (MF), was performed using ConsensusPathDB (http://cpdb.molgen.mpg.de/CPDB) (GO level 2 categories) and pathway enrichment analysis (pathways defined by KEGG and Reactome databases). The pathways were significantly enriched with a *p value* < 0.05. The online target-predicting database circMir (http://www.bio-inf.cn/circmir/) was used for the prediction of potential targeted sequences between circRNAs and miRNAs. Two additional databases, TargetScan (http://www.targetscan.org/) and miRanda (http://www.microrna.org/microrna/home.do), were used for the prediction of potential targeted sequences between miRNAs and the mRNA.

### Cell cultures

The mouse microglial cell line BV2 and mouse hippocampal neuronal cell line HT22 (ATCC, Manassas, VA) were cultured in DMEM containing 10% FBS and 1% penicillin–streptomycin solution and incubated at 37 °C in 5% CO_2_. Different combinations of lipopolysaccharide (LPS) (1 μg/mL), adenosine (10 μM), MRS1706 (0.3 μM), and MK2206 (3 μM) were added to the media 24 hours after plasmid transfection, and 8 hours later, mRNA and protein were harvested.

### Cell death assay

Then, the rates of cell death were detected by flow cytometry (Beckman Coulter, Brea, CA, USA). Briefly, cultured BV2 cells (1 × 10^5^/well) were collected at 48 h after transfection and rinsed in chilled PBS, followed by staining with a detection kit (Invitrogen) in the dark for 15 min. Next, the pellet was resuspended in 400 µL of binding buffer and stained with 5 µL of Annexin-V provided in the kit. Afterward, the cells were analyzed using a Novocyte flow cytometer (ACEA Biosciences Inc., San Diego, CA, USA).

### Enzyme-linked immunosorbent assay (ELISA)

Proinflammatory cytokines (tumor necrosis factor-α (TNF-α) and interleukin 6 (IL-6)) were measured in the culture medium of stimulated and unstimulated microglia 24 h after the last stimulation using mouse ELISA kits (Proreintech, Wuhan, China). Experiments were conducted according to the manufacturer’s protocol. Cytokine levels were measured in a plate reader at 405 nm, with wavelength correction at 650 nm. Cytokine concentrations (pg/mL) were determined using a standard calibration curve. The mean value ± SEM was established among equally treated samples. Each experiment was conducted in triplicate.

### siRNA transfection

BV2 cells were replanted 24 hours before transfection in 2 mL of fresh culture medium and cultured in growth conditions to a density of 2 × 10^5^ cells in a six-well culture plate. The cells were washed twice with PBS and then incubated in fresh DMEM containing 10% FBS after transfection. The cells were transfected with 100 pM/well siRNA according to the fast-forward protocol of the manufacturer’s instructions. Three types of siRNAs and a negative control siRNA were purchased from Sangon Biotech (Shanghai). The sequence of the siRNA is presented in Table 1. Briefly, the siRNA duplex (10 µM) was diluted in Opti-MEM® medium (Thermo Scientific), and the diluted siRNA was added to diluted Lipofectamine® RNAiMAX reagent (1:1). At room temperature, the siRNA-lipid complex was incubated for 5 min. Then, the complex was added to cells and incubated for an additional 24 h. After transfection, media was added to transfected cells to complete the media and incubated for 18 h [23].

**Table 1.** Specific RNAs primers for quantitative qRT-PCR analysis

CircRNAs	Sequence 3ʹ-5’
circ-SiRNA1	AAAGATTGCAGTATATCTTTGGCG
circ-SiRNA2	TGTAGCCATAAAGATTGCAGTATATCT
circ-SiRNA3	TTGCAGTATATCTTTGGCGATTTCA
circ-1-R	CTCAACAGAAGAGCGGGGAGTCACA
circ-2-F	GATTGCAGTATATCTTTGGCGATTT
circ-2-R	TTCATCTACACCAAACTCGATCCTC
circ-3-F	GATTGCAGTATATCTTTGGCGATTT
circ-3-R	CCAAACTCGATCCTCTGGTGCTCCT
MiR-152-R	TCCTACCGGGCCCAAGTTC
MiR-152-F	CCCAGGTTCTGTGATACACTCC
CD84-R	CTGGGTTATGGTAACTTCAGCTT
CD84-F	TCTGTGGATCTGGTTCCTTTGC
GAPDH-R	TCCCTCAAGATTGTCAGCAA
GAPDH-F	AGATCCACAACGGATACATT
U6-R	AACAAGGCTTTTCTCCAAGGG
U6-F	ACAGCACAAAAGGAAACTCACC

### Real-time quantitative PCR (RT–qPCR)

Total RNA was isolated from BV2 cells using TRIzol Reagent (TransGen Biotech). Subsequently, cDNA was synthesized from 200 ng of extracted total RNA using SuperScript III® (Invitrogen) and amplified by RT–qPCR based on the TaqMan method on an ABI PRISM 7500 Sequence Detection System (Life Technologies, Grand Island, NY, USA) with the housekeeping gene GAPDH or U6 as an internal control. The 2^−ΔΔCq^ method was used to determine the relative quantification of gene expression levels. All primer sequences were synthesized by RiboBio Co., Ltd. and are shown in Table 1.

### Dual-luciferase reporter assay

The circ-Usp10 sequence in BV2 cells was subcloned into the luciferase reporter psiCHECK2 (Promega, Madison, WI, USA) and designated psiCHECK2-circ-Usp10-WT. The circ-Usp10 sequence with mutation of the miR-152 binding site was synthesized using overlap extension PCR and cloned into the psiCHECK2 vector designated psiCHECK2-circ-Usp10-Mut. The mutant vector for the miR-152 binding site was constructed and termed psiCHECK2-miR-152-3′UTR-Mut. A total of 3 × 10^4^ BV2 cells were seeded in 24-well plates in triplicate. At 48 h following transfection with miR-152 mimics, luciferase reporter assays were conducted using the dual-luciferase reporter assay system (Promega) according to the manufacturer’s instructions. Relative luciferase activity was normalized to the Renilla luciferase internal control.

### Statistical analysis

Data are presented as the mean ± SD, and comparisons were calculated by Student’s t test by GraphPad Prism. We utilized a quartile normalization algorithm to subtract and correct the background.

## Results

In the present study, we aimed to investigate the regulatory roles of circRNAs in SCI. The SCI mouse model was used, and RNA-Seq was performed. Integrated bioinformatics analysis was used to identify DEGs and study the related pathways. Then, a circRNA-mediated ceRNA network and subnetwork were constructed, and cellular validation of related circRNAs was carried out. The details are as follow.

### The DEGs in SCI were associated with microglial/macrophage activation

**Figure 1.**
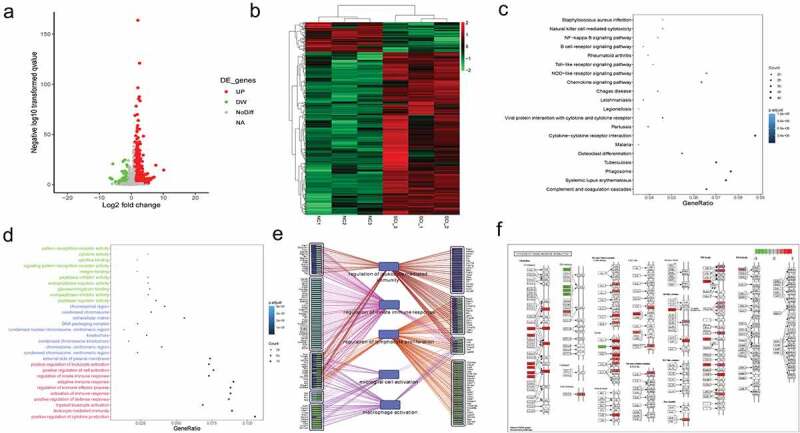
The DEGs associated with microglial/macrophage activation

First, to investigate the underlying genetic mechanism of SCI, we established a SCI mouse model and performed RNA-Seq and bioinformatics analysis. As shown in Figure 1(a,b), the volcano plot and heatmap show the 1327 DE-mRNAs in the SCI group compared with the control group. Then, KEGG pathway analysis was carried out. Signaling pathways such as the chemokine signaling, cytokine−cytokine receptor interaction, and NF−kappa B signaling pathways were significantly enriched (Figure 1(c)). In addition, the GO term analysis demonstrated that cytokine activity, cytokine binding, positive regulation of cell activation and so on were also significantly enriched (Figure 1(d)). Furthermore, we used String to construct the network and revealed that the DEGs in SCI were associated with microglial cell activation and macrophage activation (Figure 1(e)). The cytokine-cytokine receptor interaction pathway is shown in figure 1(f). Chemokines such as CCL9 and TGF-β family-like TGFB1 were upregulated.

(a-b) The volcano plot (a) and heatmap (b) of DE-mRNAs in the SCI group compared with the control group. Upregulated and downregulated genes are colored red and green, respectively. (c) The KEGG enrichment analysis of DE-mRNAs. (d) The GO enrichment analysis of DE-mRNAs. (e) Network showing the relationship of pathways and target genes. (f) The pathway of cytokine-cytokine receptor interaction rendered by KEGG.

### Identification and functional enrichment of the DE-circRNAs and DE-miRNAs

**Figure 2.**
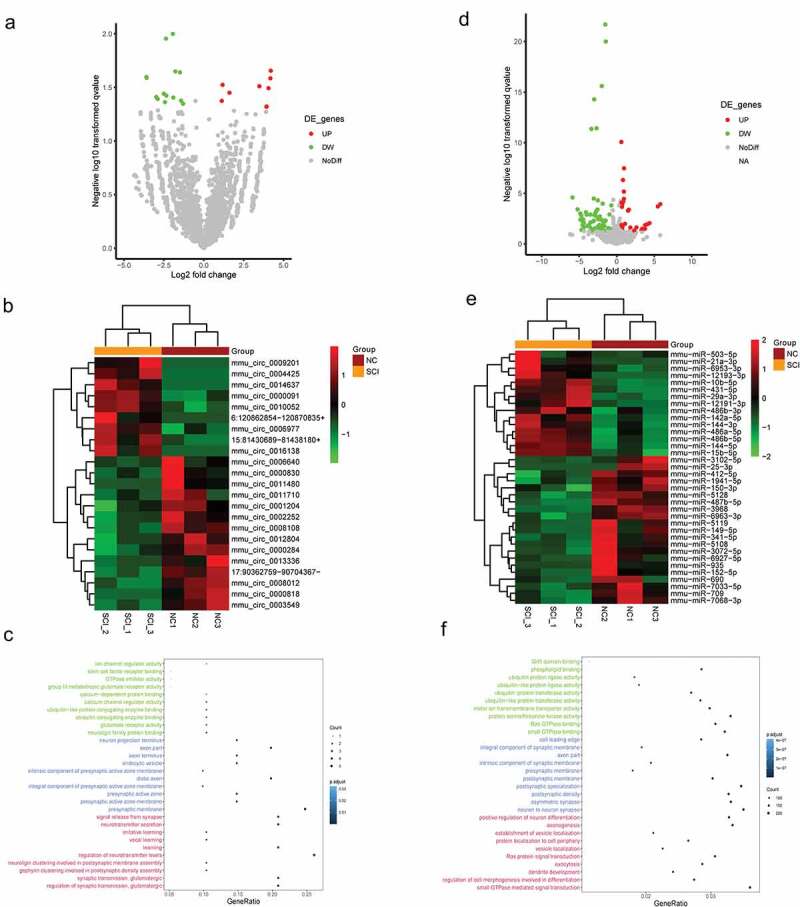
Identification and functional enrichment of the DE-circRNAs and DE-miRNAs

In the next step, we continually screened the DE-circRNAs and DE-miRNAs to lay the foundation for the construction of a circRNA-mediated ceRNA network. As presented in Figure 2(a) and Figure 2(b), a total of 23 DE-circRNAs in the SCI group compared with the control group are shown. Moreover, we carried out GO functional enrichment analysis based on the DE-circRNAs. As shown in Figure 2(c), GO terms such as neuroligin family protein binding, regulation of synaptic transmission, and neurotransmitter secretion were significantly enriched. Similarly, we identified a total of 127 DE-miRNAs and presented them in volcano plots (Figure 2(d)) and heatmaps (Figure 2(e)). In addition, the GO functional enrichment analysis indicated that positive regulation of neuron differentiation, neuron-to-neuron synapses, regulation of cell morphogenesis involved in differentiation, etc. were also enriched (figure 2(f)).

(a-b) The volcano plot (a) and heatmap (b) of DE-circRNAs in the SCI group compared with the control group. Upregulated and downregulated genes are colored red and green, respectively. (c) The GO enrichment analysis of DE-circRNAs.(d-e) The volcano plot (d) and heatmap (e) of DE-miRNAs in the SCI group compared with the control group. (f) The GO enrichment analysis of DE-miRNAs.

### Construction of the ceRNA subnetwork that regulated microglial/macrophage activation

**Figure 3.**
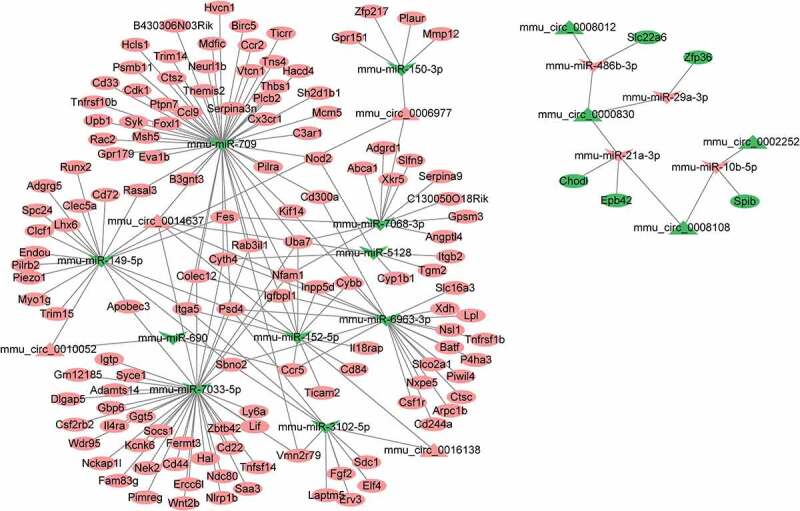
Construction of the circRNA-mediated ceRNA network

**Figure 4.**
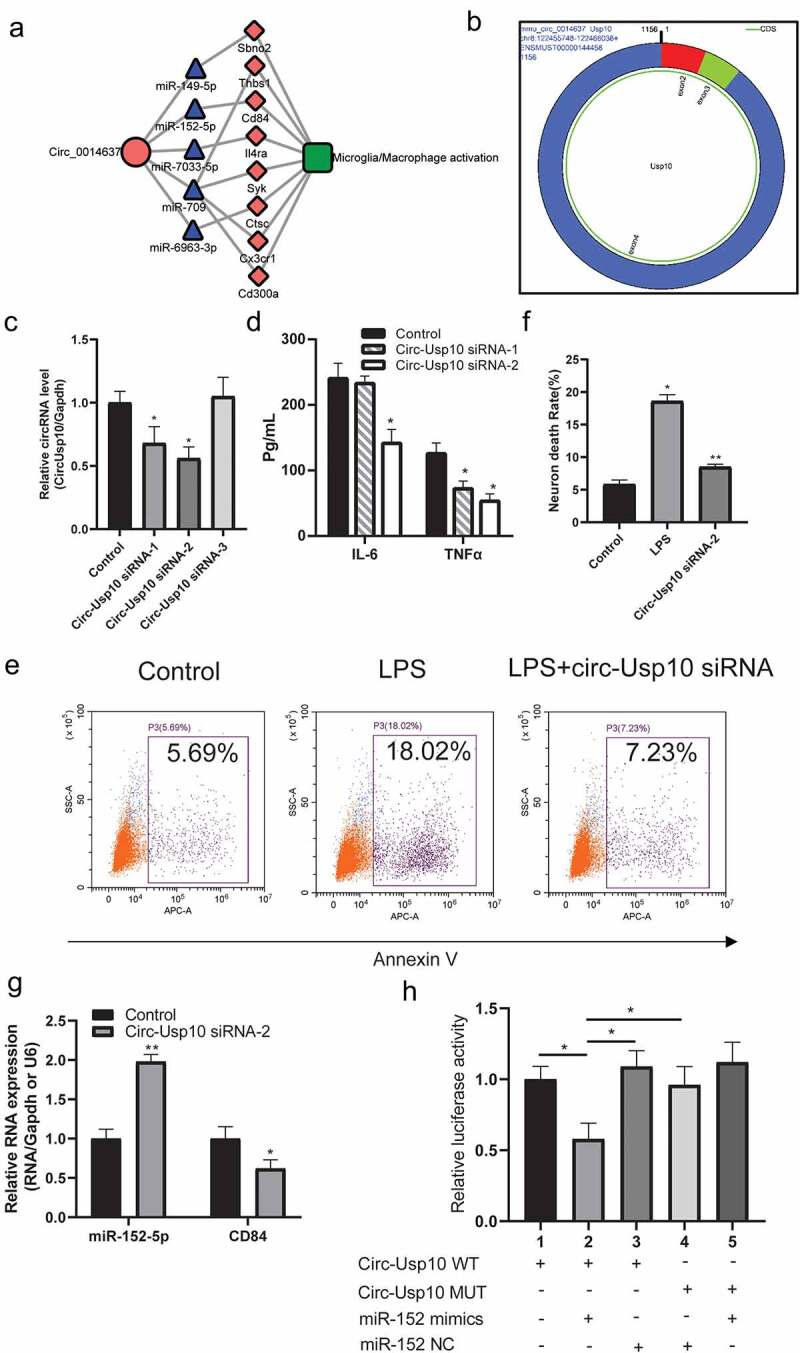
Circ-USP10 promotes microglial/macrophage activation via miR-152/CD84

To investigate the interaction between DEGs, a ceRNA network of circRNA-miRNA-mRNA was constructed according to the relationship between circRNA-miRNA and miRNA-mRNA (Figure 3). A total of 14 miRNAs (including miR-150, miR-709, miR-7068, miR-149, miR-5128, miR-690, miR-152, miR-7033, miR-3102, miR-6963, miR-486b, miR-29a, miR-21a, and miR-10b) and 8 circRNAs (including mmu_circ_0006977, mmu_circ_0014637, mmu_circ_0016138, mmu_circ_0010052, mmu_circ_0008012, mmu_circ_0000830, mmu_circ_0002252 and mmu_circ_0008108) were included in the ceRNA network. Furthermore, a subnetwork regulating microglial/macrophage activation that contained circ_0014637 (circ-Usp10), 5 miRNAs (miR-149, miR-152, miR-7033, miR-709 and miR-6963) and 8 target genes (Sbno2, Thbs1Cx3cr1, Cd300a, Il4ra, Syk, Ctsc, Cd84, and Sbno2) was further screened (Figure 4(a)). The sequencing information is presented in Figure 4(b). Nevertheless, the effect of circ-Usp10 on SCI has not yet been reported.

Construction of the circRNA-miRNA-mRNA network based on the DEGs related to SCI. The circle, triangle, and arrow indicate mRNAs, circRNAs, and miRNAs, respectively. Red indicates upregulation, and green indicates downregulation.

(a) Construction of the circRNA-miRNA-mRNA subnetwork based on circ-Usp10. The red circle, blue triangle, and red diamond indicate circRNAs, miRNAs, and mRNAs, respectively. (b) The sequence information of circ-Usp10 was downloaded from circPrimer. (c) qRT–PCR showing the relative expression levels of control, circ-Usp10 siRNA-1, circ-Usp10 siRNA-2, and circ-Usp10 siRNA-3 in BV2 cells. (d) ELISA assay showing the expression of IL-6 and TNF-α in BV-2 cells treated with normal saline, circ-Usp10 siRNA-1 and circ-Usp10 siRNA-2. (e) Representative flow cytometry showing that circ-Usp10 siRNA-2 inhibits the death of HT22 cells. (f) The neuronal rate of HT22 cells in flow cytometry experiments. (g) qRT–PCR showing the relative expression levels of miR-152 and CD84 in BV2 cells treated with normal saline or circ-Usp10 siRNA-2. (h) A dual-luciferase reporter assay was performed in BV2 cells. Results are expressed as the mean ± SEM. All experiments were repeated three times. *p < 0.05, **p < 0.01, ***p < 0.001****p < 0.0001 compared with the control group.

### Circ-Usp10 promotes microglial/macrophage activation via miR-152/CD84 in microglia

To investigate and verify the function of circ-Usp10 in SCI in vitro, we performed a cellular experiment in vitro. Three siRNAs were designed to influence the expression of circ-Usp10 in microglial BV2 cells stimulated by LPS. As shown in Figure 4(c), circ-Usp10 siRNA-1 and circ-Usp10 siRNA-2 significantly decreased the expression of circ-Usp10 in microglial BV2 cells compared to the control group. Thereafter, we found that circ-Usp10 siRNA-2 significantly inhibited the expression of the proinflammatory factors IL-6 and TNF-α in microglia treated with LPS (Figure 4(d)). Hence, we chose circ-Usp10 siRNA-2 for the following experiments. Supernatants treated with the neuronal cell line HT22 and circ-Usp10 silencing exhibited significantly induced neuronal death (Figure 4(e) and figure 4(f)). In the microglia/macrophage-activated ceRNA subnetwork, miR-152 promotes neuronal repair [24] and inhibits inflammation [25–27], and CD84 is involved in macrophage activation and the regulation of inflammatory factor secretion [28,29]. Hence, we further detected the expression of miR-152-5p and CD84 in BV2 cells. As shown in Figure 4(g), the expression of miR-152 was significantly enhanced and CD84 was significantly decreased with treatment with circ-Usp10 siRNA-2 in BV2 cells. Luciferase reporter gene experiments verified the tight binding of miR-152 and CD84 (Figure 4(h)). These results indicated that circ-Usp10 promotes microglial/macrophage activation via miR-152/CD84 in microglia.

## Discussion

As a common neurological disease that involves neuron inflammation and apoptosis pathological processes, SCI may cause long-term physical impairment and bring a substantial burden to both the individual patient and society [30,31]. Nevertheless, due to the incomplete understanding of cellular and molecular events, existing treatments for SCI are still inadequate. In this study, we focused on the differentially expressed RNAs between SCI and normal tissues, attempting to investigate the influence and mechanism of circRNAs on microglial activation and neuronal death after SCI. A mouse model of spinal cord injury was used, and 23 DE-circRNAs, 127 DE-miRNAs and 1327 DE-mRNAs were identified that were differentially expressed after spinal cord injury.

circRNAs are involved in multiple biological processes, such as acting as microRNA sponges, and are highly enriched in the nervous system with neurospecificity [32]. circRNAs not only participate in the survival and differentiation of multiple nerve cells but also promote the recovery of neurological function [33]. Accumulating evidence has revealed that circRNAs are crucial in proliferation, inflammation, and apoptosis in the central nervous system [34]. Several circRNAs have already been reported to be involved in the ceRNA network in the pathophysiological processes of neurological diseases. For example, Chen et al. suggested that circ-TYW1 serves as a sponge for microRNA-380 to accelerate neurological recovery following spinal cord injury by regulating FGF9 [35]. Liu et al. indicated that circ_HIPK3 alleviates CoCl-induced apoptotic injury in neuronal cells by regulating the miR-222-3p/DUSP19 axis [36]. Li et al. revealed that circ-FAM169A sponges miR-583, which is involved in the regulation of intervertebral disc degeneration [37]. Chen et al. demonstrated that circRNA 2960 contributes to secondary damage of spinal cord injury by sponging miRNA-124 [38]. Nevertheless, the function of circ-Usp10 in SCI is still poorly understood. Therefore, we constructed a circRNA-miRNA-mRNA ceRNA network for further investigation, and a subnetwork including the circ-Up10/miR-152/CD84 axis, which is involved in microglial/macrophage activation, was identified.

The GO enrichment analysis suggested that the DE-miRNAs were significantly enriched in neuron differentiation, neuron-to-neuron synapses, and the regulation of cell morphogenesis involved in differentiation. In the microglial/macrophage-activated ceRNA subnetwork, our results revealed a concurrent decrease in circ-Usp10 expression with elevated expression of miR-152 in BV2 cells. A previous study indicated that miR-152 promotes neuronal repair [24] and inhibits inflammation [25–27]. miR-152 can inhibit inflammatory responses and promote the recovery of spinal cord injury through the c-jun N-terminal kinase pathway and can be a target molecule for treating spinal cord injury [24]. In addition, miR-152 inhibits neuronal axon growth after spinal cord injury by regulating the p38 MAPK signaling pathway [39]. The evidence above suggests the crucial role of miR-152 in microglial/macrophage activation in SCI.

We also found that circ-Usp10 regulated the expression of the inflammation-related gene CD84. CD84 is involved in macrophage activation and the regulation of inflammatory factor secretion [28,29]. Mouse CD84 is a pan-leukocyte receptor that can modulate signaling pathways downstream of TLR4 and regulates macrophage cell fate decisions and effector functions. It was reported that the presence of CD84 increased the LPS-induced secretion of TNF-α and MCP-1 but significantly lowered IL-10 and IL-6 production. Mouse CD84 is a pan-leukocyte receptor that can modulate signaling pathways downstream of TLR4 and regulates macrophage cell fate decisions and effector functions [28]. These results suggest that circ-Usp10 may be used as a miR-152 sponge to regulate CD84 expression.

## Conclusion

In conclusion, we used a mouse model of spinal cord injury for RNA-Seq and integrated bioinformatics analysis. We identified the DE-mRNAs, DE-circRNAs and DE-miRNAs for the construction of a circRNA-mediated ceRNA network. The subnetwork including circ-Usp10/miR-152-5p/CD84, which functions in microglial activation, was screened for cellular validation. The circ-Usp10 significantly induced neuronal death. A concurrent decrease in circ-Usp10 expression occurred with elevated expression of miR-152 and a decrease in CD84 in BV2 cells. These results revealed that circ-Usp10 promoted microglial activation and induced neuronal death by targeting miR-152-5p/CD84, which might provide a new potential therapy for SCI.

## Data Availability

All datasets generated for this study are included in the article/supplementary material.
